# Comparative Evaluation of Bandit-Style Heuristic Policies for Moving Target Detection in a Linear Grid Environment

**DOI:** 10.3390/s26010226

**Published:** 2025-12-29

**Authors:** Hyunmin Kang, Minho Ahn, Yongduek Seo

**Affiliations:** 1 Digital Healthcare Center, Gumi Electronics & Information Technology Research Institute, Gumi 39253, Republic of Korea; khm@geri.re.kr; 2 Department of Artificial Intelligence, Sogang University, Seoul 04107, Republic of Korea; 3 Artificial Intelligence Laboratory, Konan Technology Inc., Seoul 06627, Republic of Korea; minho.ahn@konantech.com; 4 Department of Defense Acquisition, Konkuk University, Seoul 05209, Republic of Korea

**Keywords:** moving target detection, partially observable Markov decision process (POMDP), restless-bandit motivation (RMAB-inspired), belief-proportional sampling (BPS, probability matching), belief state, reinforcement-learning–inspired heuristics, expected time to detection (ETTD)

## Abstract

Moving-target detection under strict sensing constraints is a recurring subproblem in surveillance, search-and-rescue, and autonomous robotics. We study a canonical one-dimensional finite grid in which a sensor probes one location per time step with binary observations while the target follows reflecting random-walk dynamics. The objective is to minimize the expected time to detection using transparent, training-free decision rules defined on the belief state of the target location. We compare two belief-driven heuristics with purely online implementation: a greedy rule that always probes the most probable location and a belief-proportional sampling (BPS, probability matching) rule that samples sensing locations according to the belief distribution (i.e., posterior probability of the target location). Repeated Monte Carlo simulations quantify the exploitation–exploration trade-off and provide a self-comparison between the two policies. Across tested grid sizes, the greedy policy consistently yields the shortest expected time to detection, improving by roughly 17–20% over BPS and uniform random probing in representative settings. BPS trades some average efficiency for stochastic exploration, which can be beneficial under model mismatch. This study provides an interpretable baseline and quantitative reference for extensions to noisy sensing and higher-dimensional search.

## 1. Introduction

Moving target detection is of critical importance in domains such as defense, disaster response, and surveillance robotics, where platforms must localize potentially hostile or vulnerable objects under severe sensing constraints. In this paper, however, we do not attempt to model a full operational system in these domains. Instead, we study a stylized one-dimensional linear-grid setting that abstracts a basic sensing subtask, for example, repeatedly scanning along a corridor, border segment, or pipeline, in order to isolate core algorithmic trade-offs while keeping the model analytically and computationally tractable. Although real operational environments are inherently two- or three-dimensional, many deployed systems execute a search along preplanned one-dimensional paths, such as patrol routes, road segments, or scan lines generated by a higher-level planner. Our linear grid can thus be interpreted as an abstraction of a single such sweep, whose behavior can later be embedded into a broader 2D/3D coverage strategy. Within this simplified setting, the agent must locate the target quickly, an issue that has also been studied in classical search theory. In particular, when the probabilistic distribution of the target is known, it is well established that sequentially searching from the most likely locations yields an optimal strategy [1]. For example, in a static-target environment, probing the location with the highest probability maximizes the expected detection probability [1]. In dynamic settings, ref. [2] proposed related strategies from the perspective of dynamic hypothesis testing. However, these traditional approaches generally assume that the target’s motion model is known a priori and that the search path can be planned deterministically in advance.

In recent years, research has expanded beyond offline search planning on known maps to include real-time online pursuit-evasion using mobile robots and unmanned aerial vehicles (UAVs). In addition, there has been growing interest in approximating partially observable Markov decision process (POMDP) problems with deep reinforcement learning. For example, ref. [3] compared a deep-RL solver with traditional point-based POMDP algorithms on an “olfactory search” POMDP and showed that lightweight policies learned by deep RL can be competitive. For rapid anomaly detection under limited resources, ref. [4] proposed an active hypothesis-testing framework based on the ref. [5,6] suggested a strategy that tracks a moving target on a graph by probing the second-most-likely location. Distinct from these prior studies, this work analyzes simple bandit-style heuristic policies, inspired by reinforcement-learning and restless-bandit theory, in a stylized moving-target detection setting and evaluates them with the objective of minimizing the expected time to detection (ETTD). Rather than implementing any explicit learning algorithm, we focus on closed-form online decision rules that can be executed directly on the belief state without computing a complex optimal policy. The central scientific question of this study is as follows: to what extent can simple, interpretable, training-free belief-based heuristics serve as effective online baselines for moving-target detection under strict sensing constraints? Specifically, we address three research questions:

(RQ1) How do greedy (maximum-belief) probing and belief-proportional probing differ in ETTD and in the overall detection-time distribution under the reflecting random-walk model?
(RQ2) What exploitation–exploration trade-off emerges between greedy (maximum-belief probing) and BPS (belief-proportional sampling), and when can stochastic exploration improve robustness (e.g., reduced sensitivity to local belief peaks or model mismatch)?
(RQ3) How do the comparative trends scale with the grid size, and what quantitative performance gaps can be established as a reusable baseline for future extensions?

The remainder of this paper is organized as follows: Section 2 reviews related work, followed by the problem definition in Section 3. Section 4 introduces the belief update mechanism and the decision rules for greedy and BPS policies. Section 5 analyzes their performance through simulations, and Section 6 discusses the results. Finally, Section 7 concludes with directions for future research. The specific contributions of this paper are summarized as follows:

1. We formulate single-target moving-target detection in a one-dimensional reflecting random-walk grid as a POMDP over a belief state, and we adopt the ETTD as the primary performance metric.
2. We present two training-free, purely online heuristic policies with closed-form decision rules—(i) greedy (maximum-belief probing) and (ii) belief-proportional sampling (BPS)—and we benchmark them against two simple baselines: ε-greedy (controlled exploration) and uniform random probing.
3. Under the stylized 1D model, we provide an analytic characterization and interpretation of the two policies’ ETTD behavior, connecting the insights to classical sequential search and bandit-inspired decision-making.
4. We conduct systematic simulations across grid sizes to quantify ETTD improvements and detection-time distributions, providing a transparent baseline for future extensions to noisy sensors and higher-dimensional settings.

## 2. Related Work

The problems of searching for and tracking moving targets have been studied for a long time using a variety of approaches. Traditionally, search plans have been proposed to maximize the probability of detection within a finite time horizon for both static and moving targets [1,7]. For example, strategies that improve the efficiency of sequential search for static or moving targets have been presented in [1,7]; these studies typically adopt a planning-based approach that models target motion as a Markov chain.

In robotics, alongside offline path planning that exploits known maps, online strategies have been proposed to track targets in real time within the sensor’s field of view. A survey of search/pursuit problems for mobile robots and a taxonomy of scenarios is provided in [8], while efficient path planning in indoor environments with multiple robots is studied in [9]. They showed that, given a known map, the robot path-selection problem is NP-hard and proposed scalable approximation algorithms based on finite-horizon planning and coordinated cooperation [9]. In contrast, when map information is unavailable, research has addressed cooperative search with multiple UAVs and optimal resource allocation. For cases in which map information is unavailable, Bayesian estimation-based cooperative UAV search has been proposed in [10], and a recursive Bayesian search-and-tracking algorithm using coordinated UAVs is presented in [11]. These techniques update the probability distribution of the target’s location in real time, thereby improving search strategies even under limited sensing resources.

Recent years have also seen active research using reinforcement learning (RL). A deep reinforcement-learning approximation for an olfactory-search partially observable Markov decision process is compared with point-based methods in [3]. For multi-UAV cooperative search in three-dimensional environments, multi-agent learning-based cooperative search in three-dimensional settings is investigated in [12,13,14]. Collectively, these studies extend RL-based search problems to 2D/3D spaces, multi-agent settings, and dynamically moving targets, demonstrating the utility of learning-based approximations in lieu of solving the full POMDP exactly [15]. Beyond these domain-specific applications, recurrent deep RL architectures such as deep recurrent Q-networks (DRQN), asynchronous advantage actor–critic with long short-term memory (A3C-LSTM), and proximal policy optimization (PPO) have been proposed to maintain an internal memory of past observations, making them particularly suitable for POMDP settings. In this context, the present work does not aim to compete with such high-capacity controllers, but rather to provide a lightweight, analytically transparent baseline against which more expressive recurrent deep RL policies can be evaluated in future moving-target detection studies. In contrast to these deep recurrent approaches, the present work focuses on simple, closed-form policies (greedy and belief-proportional sampling) without any parameter learning, using them as interpretable reference baselines rather than as competitors to state-of-the-art deep RL controllers. Given the very high computational complexity of obtaining exact POMDP optima [16], the present work offers a new perspective based on approximate online learning. Related work includes analyses of why certain POMDPs are easier to approximate [17], Monte Carlo value iteration for continuous-state settings [18], and point-based algorithms such as Successive Approximations of the Reachable Space under Optimal Policies (SARSOPs) and related advances [19,20,21,22,23]. Likewise, indexability and bandit-theoretic analyses relevant to exploration are discussed in [24,25], and a general reinforcement-learning perspective is summarized in [26].

Finally, as noted in [26], learning-based approaches can serve as powerful alternatives even for complex decision-making problems. To clarify how our study relates to prior work and to highlight the motivation for our approach, Table 1 summarizes representative lines of research, along with their key advantages and limitations with respect to online moving-target detection under sensing constraints. This comparison helps identify the gap addressed by this paper: an analytically transparent, training-free baseline that bridges classical search theory and bandit-style sequential decision-making while directly targeting expected time to detection.

As summarized in Table 1, prior work spans classical search theory, robotics planning, Bayesian multi-agent tracking, POMDP solvers, and deep RL. However, there remains a practical need for a simple, interpretable, and computationally lightweight benchmark that can be executed fully online without a training phase, while still capturing key POMDP characteristics through belief updates. Motivated by this gap, we focus on two closed-form bandit-style heuristics, greedy sensing based on the maximum belief and stochastic sensing based on belief-proportional sampling, and evaluate their trade-offs using ETTD as the primary metric.

## 3. Problem Definition

In this work, the environment is a one-dimensional grid composed of N locations (slots) arranged linearly. The slots are connected in a line, where slot 1 and slot N correspond to the left and right boundaries of the grid, respectively. A single moving target exists on the grid and, at each discrete time step t=0,1,2…, occupies exactly one slot. Let $X_{t}\in S$ denote the discrete target position at time t, where the finite state space (sample space) is S={1,2,…,N}. The target motion is modeled as a time-homogeneous Markov chain with transition matrix T, whose entries are defined by $T_{ji}≜P(X_{t+1}=j|X_{t}=i),i,j\in S.$ Because $X_{t}$ takes values on a finite, discrete set, all location distributions (including the belief state $b_{t}$) are probability mass functions (PMFs) over S (i.e., categorical distributions), rather than probability density functions (PDFs), which are used for continuous-valued random variables.

From an application viewpoint, such a one-dimensional grid can represent a discretized track along which a UAV, ground vehicle, or pan-tilt sensor repeatedly scans, for example, a corridor in a building, a pipeline or power line, a perimeter fence, or a coastline segment. In realistic two- or three-dimensional missions, a higher-level planner could decompose the workspace into a sequence of such linear tracks, and the policies analyzed in this paper would then govern the allocation of sensing effort along each track. In this sense, the present model should be viewed as a building block for larger 2D/3D search-and-tracking systems, rather than a full description of the entire operational space. The target’s motion model is given by a random walk as follows:

- Interior slot (2≤i≤N−1): at the next time step, the target moves to the left (i−1), stays (i), or moves to the right (i+1) with equal probability $\frac{1}{3}$.
- Left boundary (slot 1): at the next time step, the target remains at the boundary with probability $\frac{2}{3}$ or moves to the right (slot 2) with probability $\frac{1}{3}$.
- Right boundary (slot N): at the next time step, the target remains at the boundary with probability $\frac{2}{3}$ or moves to the left (slot N−1) with probability $\frac{1}{3}$.

These boundary probabilities (2/3 for staying and 1/3 for moving inward) are chosen to define a simple reflecting random walk that preserves the symmetry of the interior motion while enforcing hard boundaries on the finite grid. In the interior, the target has three symmetric options (move left, stay, or move right), and each is assigned a probability of 1/3. Formally, we start from the interior kernel $P(X_{t+1}=i-1|X_{t}=i)=P(X_{t+1}=i|X_{t}=i)=P(X_{t+1}=i+1|X_{t}=i)=1/3$. At the left boundary i=1, the inadmissible outcome $X_{t+1}=0\notin S$ would receive probability mass 1/3; we fold (reassign) that mass onto the admissible “stay” event, giving $P(X_{t+1}=1|X_{t}=1)=1/3+1/3=2/3$ and $P(X_{t+1}=2|X_{t}=1)=1/3$ (analogously for i=N). At a physical boundary, the “outside” location is not admissible, so the probability mass that would have exited the domain is reassigned to the stay event, yielding a 2/3 chance of remaining at the boundary and a 1/3 chance of returning toward the interior. This construction leads to an ergodic Markov chain with nearly uniform stationary behavior and avoids introducing an artificial drift either toward or away from the boundaries. Similar reflecting random-walk models are widely used as minimal abstractions for diffusive motion in bounded domains in the search-theoretic literature [1,7]. While this equal-probability random walk is clearly a simplification relative to real targets, which may exhibit directional drift, dwell-time asymmetries, patrol patterns, or adversarial evasive maneuvers, we adopt it here as a canonical, symmetric baseline that isolates the effect of the sensing policy from higher-level motion planning. More general Markovian or semi-Markov motion models can be accommodated in the same framework by replacing the transition matrix T with any stochastic matrix capturing the desired dynamics; the belief-propagation step $b_{t+1}^{\prime }=b_{t}\times T$ in Equation (4) and the greedy/BPS policies then remain unchanged. Under this model, the state transition of the target is represented by the transition matrix T, where $P\left(X_{t+1}=j|X_{t}=i\right)=T_{ji}$, and the entries are summarized in Table 2.

Sensors (agents) select one slot s∈{1,…,N} on the grid at each time step and can send a sensing signal to that location. The sensing outcome is a binary observation o∈{0,1}: if the target is present in the chosen slot, then o=1 (detection), otherwise, o=0 (miss). In this study, we assume an idealized binary sensor with perfect detection (probability of detection $P_{d}=1$) and no false alarms (probability of false alarm $P_{fa}=0$), so that the observation depends only on whether the target truly occupies the sensed slot. This assumption is adopted to isolate the effects of the motion model and the belief-update mechanism and to keep the analysis analytically tractable. In practical systems, $0<P_{d}<1$ and $0<P_{fa}<1$ would induce a noisy observation model; the belief-update framework in Section 4.1 can be generalized by replacing the deterministic rule in Equation (5) with a Bayesian update that incorporates the likelihood $P(o_{t}|X_{t},s_{t})$ under given $P_{d}$ and $P_{fa}$. A quantitative study of specific sensor-noise parameters is left to future work. In addition to this idealized setting, Section 5.5 presents a sensitivity study (see Section 5.5), in which the same policies are evaluated under a simple noisy-sensor model with $P_{d}=0.8$ and $P_{fa}=0.02$ in order to illustrate how reduced detection probability and low-rate false alarms qualitatively affect the ETTD. These values were chosen to represent a moderate-miss, low-false-alarm operating point for a thresholded binary detector: $P_{d}=0.8$ corresponds to a 20% miss probability, while $P_{fa}=0.02$ introduces rare but non-negligible spurious alarms. This choice allows us to test robustness to both error modes without letting false-alarm terminations dominate the alarm-time statistic.

In Section 3, Section 4, Section 4.1, Section 4.2, Section 4.3, Section 4.4, Section 5, Section 5.1, Section 5.2, Section 5.3 and Section 5.4, the ideal sensor setting ($P_{d}=1,\;\;P_{fa}=0$) makes an alarm (o=1) equivalent to a true detection. In the sensitivity study with false alarms (Section 5.5), we keep the same stopping rule (terminate on the first alarm); therefore, $T_{D}$ in that subsection should be interpreted as time-to-first-alarm (true or false), i.e., an “alarm time” rather than a guaranteed time-to-true-detection. During a single sensing action, the target moves once before the result is observed; so, the observation $o_{t}$ obtained after sensing slot s at time t can be regarded as a signal indicating whether the target is in that slot after one transition (t−1→t). When detection occurs (o=1), the algorithm terminates immediately and the time $T_{D}$ taken to complete detection is recorded; each sensing action incurs the same unit cost (or unit time) 1. The objective of this study is therefore to minimize the ETTD, i.e., $E_{\pi }[T_{D}]$. In other words, the optimal policy is the one that finds the target with as few sensing steps as possible. The agent does not know the target’s initial position exactly and relies only on past sensing observations to make a probabilistic estimate of the target’s location, namely, the belief state. We denote the belief at time t by $b_{t}=[b_{t}\left(1\right),\;b_{t}\left(2\right),\ldots ,b_{t}\left(N\right)]$, where $b_{t}\left(i\right)=P(X_{t}=i|history)$. Here, “history” refers to all actions (chosen sensing slots) and all observations up to time t; thus, $b_{t}$ is the posterior induced by the cumulative evidence. Since $b_{t}$ is a probability distribution over all slots, $\sum_{i=1}^{N}b_{t}(i)=1$. The initial belief $b_{0}$ depends on prior information; if the initial position of the target is completely unknown, we set $b_{0}\left(i\right)=\frac{1}{N}$.

In summary, the problem can be viewed as a partially observable control task with state ($X_{t},b_{t}$), in which the agent selects a sensing slot $s_{t}$ as the action and receives an observation $o_{t}$. The target state $X_{t}$ is hidden, while the belief state $b_{t}$ serves as the observable state in a belief-MDP framework perceived by the agent. The agent chooses $s_{t}$ according to a policy π, and the goal is to find a policy $\pi^{*}$ that minimizes the ETTD, i.e., $E_{\pi }[T_{D}]$. In general, this objective is equivalent to a POMDP cost-minimization problem with unit stage cost, for which the optimal policy can, in principle, be obtained by dynamic programming over the belief space; however, due to computational intractability, approximate or heuristic approaches are required. In the next section, we describe the two proposed belief-driven heuristic policies (greedy and BPS) together with their belief-update procedures and action-selection principles.

## 4. Methodology

In this study, we employ two bandit-style heuristic policies, inspired by reinforcement-learning ideas, as approximations to the optimal policy:

1. RMAB-inspired greedy heuristic (belief-argmax policy)—at each time step, sense the slot with the highest probability of containing the target under the current belief state.
2. Belief-proportional sampling (BPS; probability-matching) policy—sample a slot from the current belief distribution (interpreted as a probability model of the target’s location) and sense that slot.

These policies are defined analytically in terms of the current belief state and do not undergo any separate training or parameter update; in this sense, our use of the term “reinforcement-learning-inspired” refers to the sequential decision-making and exploration–exploitation concepts rather than to a learned value function or policy network.

### 4.1. Belief-State Update

The agent performs the following cyclic procedure at every time step: (i) it predicts the target’s motion and propagates the belief state; (ii) it determines the location to search, deploys the sensor, and obtains an observation; and (iii) it updates the belief by reflecting the observation result (in case of detection or miss). Described more formally, it is as follows:

1. Prediction: Apply the target’s transition–probability matrix T (the N×N transition matrix corresponding to Table 2) to the belief $b_{t}$ at time t to compute the prior belief $b_{t+1}^{\prime }$ at the next time. Then, $b_{t+1}^{\prime }=b_{t}\times T$, and elementwise it is given by Equation (4) below:
  (4) $$b_{t+1}^{\prime }(j)=\sum_{i=1}^{N}P(X_{t+1}=j|X_{t}=i)b_{t}(i)$$

Equation (4) is the predicted one-step-ahead location distribution obtained by taking into account the target location distribution $b_{t}$ at time t.

2. Sensing action: According to the predicted distribution $b_{t+1}^{\prime }$, the sensor selects a slot $s^{*}$ to search. This selection depends on the policy, and the concrete selection criteria for the two proposed policies (greedy and BPS) are explained in the subsequent subsections. At the selected slot $s^{*}$, the sensor emits a pulse to check for the presence of the target.
3. Observation and termination condition: The sensor checks the observation result o. If o=detection (i.e., the target is in the selected slot $s^{*}$ and is captured by the sensor), then the target has been found, the objective is achieved, and the process terminates. At this time, the detection time is recorded, and the overall algorithm ends. In contrast, if o=miss (the selected slot did not contain the target), the process continues because the target has not been found.
4. Update: When detection fails, we obtain information that there is no target in the probed slot $s^{*}$. Therefore, in $b_{t+1}^{\prime }$, we set the probability mass of slot $s^{*}$ to 0 and compute the posterior belief $b_{t+1}$ by renormalizing the remaining probabilities. This yields Equation (5):
  (5) $$b_{t+1}\left(s^{*}\right)=0,\;b_{t+1}\left(i\right)=\frac{b_{t+1}^{\prime }(i)}{1-b_{t+1}^{\prime }(s^{*})}\;(i\neq s^{*})$$

That is, Equation (5) removes the prior probability mass $b_{t+1}^{\prime }(s^{*})$ of the selected slot $s^{*}$ and then divides the remaining probabilities by $1-b_{t+1}^{\prime }(s^{*})$ so that they sum to 1. The resulting $b_{t+1}$ becomes the new belief state at time t+1, after which we increase t←t+1 and repeat the above procedure.

**Figure 1:**
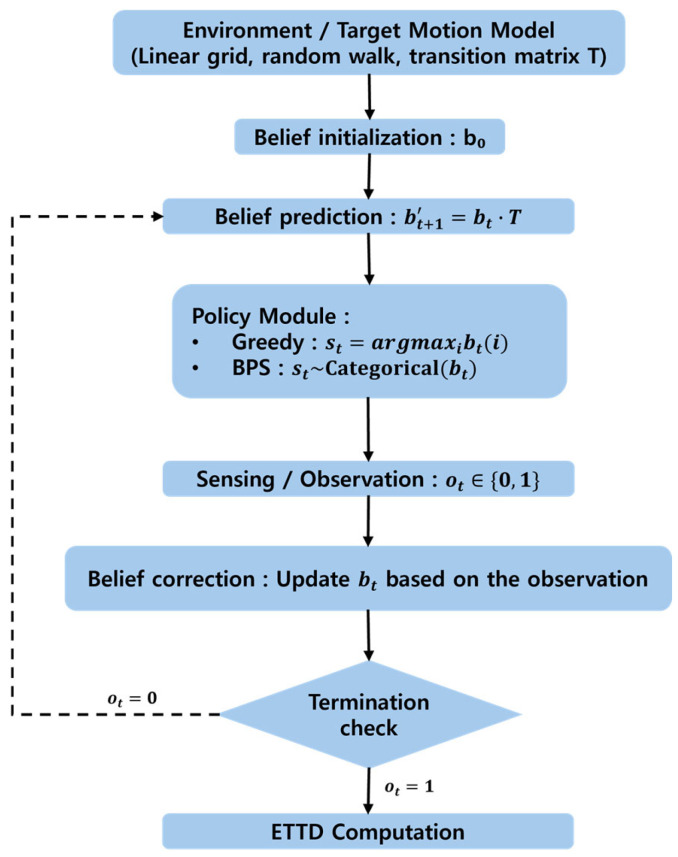
Belief-state prediction and update flowchart.

The above procedure is repeated at every time step until o=1 (detection). Figure 1 presents this belief-state update procedure as a flowchart. In this process, only the action selection of step 2 differs depending on the agent’s policy, while the remaining prediction/update steps and the Bayesian update operate under the same principle.

### 4.2. RMAB-Inspired Greedy Heuristic (Belief-Argmax Policy)

The RMAB problem is a generalization of the multi-armed bandit (MAB) in which the state of each arm evolves over time even when the arm is not played. In our target detection setting, this corresponds to the restless situation where the target’s state (location probabilities) changes over time, regardless of which slot is sensed. Ref. [6] proposed the Whittle index as a rule for computing near-optimal policies for such problems; however, computing indices for general RMABs requires additional analysis and can be expensive. In this study, instead of computing Whittle indices, we adopt a simple heuristic that always probes the slot with the highest probability. Accordingly, we refer to this policy as RMAB-inspired rather than a Whittle index RMAB policy.

The greedy heuristic policy $\pi_{\mathrm{greedy}}$ selects, at every step, the slot with the highest probability under the current belief distribution and then senses that slot. Formally, the action at time t+1 is given by Equation (6):

(6) $$s_{t+1}=\arg\underset{i}{\max}\;b_{t+1}^{\prime }\left(i\right)$$

This strategy immediately probes the slot that is currently most likely to contain the target, thus maximizing the one-step detection probability $\underset{i}{\max}b_{t+1}^{\prime }\left(i\right)$. The computation is very simple: at each time step it suffices to take the maximum over N values, yielding time complexity O(N). More generally, one full iteration of the belief-update loop consists of three basic operations: (i) prediction via the matrix–vector multiplication $b_{t+1}^{\prime }=b_{t}T$; (ii) selection of the sensing slot according to the policy (argmax for the greedy rule or belief-proportional sampling); and (iii) renormalization of the belief according to Equation (5). For a dense transition matrix T, the prediction step requires $O(N^{2})$ operations, while the argmax/sampling and renormalization each require O(N) operations, so the overall per-step cost is $O(N^{2})$. If the random walk is local and T is implemented as a sparse banded matrix with a constant number of nonzero entries per column, the per-step cost reduces to O(N). For an episode that stops after $T_{D}$ sensing steps, the total computational cost therefore scales as $O(T_{D}N^{2})$ in the dense case and $O(T_{D}N)$ in the sparse case, which is modest for the grid sizes and detection times considered in Section 5. In the static-target case (i.e., the target does not move), a greedy policy that sequentially probes the slot with the largest probability mass in the initial belief $b_{0}$—that is, $\underset{i}{\max}\;b_{0}\left(i\right)$—achieves (near) minimal expected detection time. The success probability at the first step is simply $\underset{i}{\max}\;b_{0}\left(i\right)$; under a geometric approximation, this yields Equation (7):

(7) $$\left[T_{\pi_{greedy}}\right]≲\frac{1}{\underset{i}{\max}\;b_{0}\left(i\right)}$$

Accordingly, for a static target the greedy policy is close to optimal. In contrast, when the target moves, the greedy policy can overfit the prior and keep probing an incorrect location. In particular, if we assume the target tends to move in the opposite direction, the agent may end up perpetually “chasing the tail,” producing worst-case detection time that scales with the grid size N, i.e., O(N). In such cases the policy may get stuck in a local optimum, failing to explore the entire space. That said, in our stochastic random-walk setting the probability of such adversarial persistence is very low; empirically, the greedy heuristic exhibited superior average performance in most scenarios.

From a computational perspective, the greedy update in the present one-dimensional grid requires only an O(N) scan over the belief vector at each step. In higher-dimensional grids obtained by discretizing a 2D or 3D workspace, a straightforward extension of the same rule would still scale linearly in the number of cells, but the resulting belief map often contains multiple spatially separated modes. In such settings, a purely myopic argmax rule can become trapped in a single high-probability region and fail to allocate sensing effort to other promising areas unless it is embedded in a higher-level spatial decomposition (e.g., row/column sweeps, sectorization) or complemented by exploratory mechanisms such as belief-proportional sampling (probability-matching). Because the appropriate decomposition and coordination strategy are highly application-dependent, we deliberately restrict the quantitative analysis in this paper to the one-dimensional case and leave a systematic complexity and scalability study for 2D/3D domains to future work.

### 4.3. Belief-Proportional Sampling (Probability-Matching)

In the standard multi-armed bandit setting, belief-proportional sampling (probability-matching) selects actions by sampling unknown model parameters from their posterior distribution and then chooses the action that is optimal under the sampled model. In our setting, there are no unknown reward parameters to sample; instead, the key uncertainty is the latent target location encoded by the belief state. Therefore, we adopt a belief-proportional sampling (probability-matching) rule: we interpret the predicted belief $b_{t+1}^{\prime }$ as a categorical distribution over locations and sample the next sensing slot directly from this belief distribution.

Equation (8) interprets the prior belief $b_{t+1}^{\prime }$ as a categorical distribution and specifies the rule for choosing the sensing location at the next time step. Here, $b_{t+1}^{\prime }\left(i\right)$ is the probability, just before observation, that the target is in slot i; using this value directly as the action probability makes high-probability slots more frequently selected while still probing low-probability slots with a nonzero chance. In other words, the policy automatically balances exploitation and exploration, imposing selection bias according to the concentration of the belief while never excluding any region entirely:

(8) $$\mathrm{Pr}\left(s_{t+1}=i\right)=b_{t+1}^{\prime }\left(i\right),\;\left(i=1,\ldots ,N\right)$$

Equation (9) gives the one-step detection success probability. It can be interpreted as the sum over events in which the target’s actual location is i and, simultaneously, slot i is selected. From the prior belief, $\mathrm{Pr}\left(X_{t+1}=i\right)=b_{t+1}^{\prime }\left(i\right)$, and from the action rule, $\mathrm{Pr}\left(s_{t+1}=i\right)=b_{t+1}^{\prime }\left(i\right)$; hence the joint probability that both occur is $b_{t+1}^{\prime }\left(i\right)\times b_{t+1}^{\prime }\left(i\right)={\left(b_{t+1}^{\prime }\left(i\right)\right)}^{2}$. Summing over all i yields the following:

(9) $$P\left(o_{t+1}=1\right)=\sum_{i=1}^{N}{(b}_{t+1}^{\prime }{\left(i\right))}^{2}$$

Therefore, as the belief becomes concentrated on a particular slot, the squared sum increases and the one-step success probability rises; conversely, a dispersed belief reduces the squared sum and lowers the success probability.

Equation (10) considers a static target, where the prior belief does not change over time. In this case, the one-step success probability for BPS is the constant $p=\sum_{i=1}^{N}b_{0}{\left(i\right)}^{2}$, and under a geometric approximation the expected number of steps until success equals $E[T]=\frac{1}{p}$. Consequently, when the initial belief mass is concentrated on a single slot, $\sum_{i=1}^{N}b_{0}{\left(i\right)}^{2}$ becomes large and the ETTD becomes short; when the belief is uniform, $\sum_{i=1}^{N}b_{0}{\left(i\right)}^{2}$ becomes small and the ETTD becomes long:

(10) $$E\left[T\pi_{\mathrm{BPS}}\right]\approx \frac{1}{\sum_{i=1}^{N}b_{0}{\left(i\right)}^{2}}$$

Equation (11) states a basic inequality for the probability vector $b_{0}$: the sum of squares $\sum_{i}b_{0}{\left(i\right)}^{2}$ is always bounded above by the maximum component $\underset{i}{\max}b_{0}\left(i\right)$. This follows because, for every i, we have $b_{0}{\left(i\right)}^{2}\leq (\underset{j}{\max}b_{0}\left(j\right))b_{0}(i)$; summing over i yields $\sum_{i}b_{0}{\left(i\right)}^{2}\leq (\underset{i}{\max}b_{0}\left(i\right))\sum_{i=1}^{N}b_{0}(i)=\underset{i}{\max}b_{0}\left(i\right)$. Equality holds only for a one-hot distribution in which all mass is concentrated on a single slot:

(11) $$\sum_{i}b_{0}{\left(i\right)}^{2}\leq \underset{i}{\max}b_{0}\left(i\right)$$

Equation (12) presents, for the static-target setting, the relationship that ETTD under BPS is greater than or equal to that of the greedy policy. The greedy policy’s one-step success probability is the largest component ${max}_{i}b_{0}(i)$, whereas for BPS it is $\sum_{i}b_{0}{\left(i\right)}^{2}$. By Equation (11), $\sum_{i}b_{0}{\left(i\right)}^{2}\leq \underset{i}{\max}b_{0}\left(i\right)$ holds. Since ETTD is the reciprocal of the success probability, we obtain the following:

(12) $$E\left[T_{\pi_{BPS}}\right]≳\frac{1}{{max}_{i}b_{0}(i)}\approx E\left[T_{\pi_{\mathrm{greedy}}}\right]$$

Thus, on average, the greedy policy is at least as favorable as BPS for a static target. By contrast, BPS continues to probe low-probability slots with a small but nonzero chance, which provides robustness and mitigates worst-case behavior when the initial belief is inaccurate. In short, for a static target the greedy policy achieves equal or shorter expected time to detection than BPS. However, BPS becomes advantageous when the target moves or when the initial distribution is broad and uncertain. Because BPS performs probabilistic exploration of lower-probability slots, it can prevent the agent from getting trapped by a purely exploitative strategy. Consequently, while BPS may be slightly less efficient on average (as also seen in our experiments), it tends to be more resilient to worst-case conditions and non-stationarity. In particular, under stochastic or adversarial motion models, the probability of eventual detection can benefit from BPS exploratory behavior. In terms of computational cost, however, BPS has the same order of complexity per step as the greedy policy, since it shares the same belief-prediction and renormalization operations and differs only in the way the sensing slot is chosen. Although both policies are suboptimal from the perspective of a Markov decision process (MDP), they can also be interpreted as index or bandit policies. The greedy policy assigns to each slot an index equal to the current probability of target presence and selects the slot with the maximum value, whereas BPS uses those probabilities to perform weighted randomization. The former’s index is not the Whittle index, but it is simple to compute and fits well with our cost structure (termination upon detection; unit cost for a miss). BPS, on the other hand, does not compute an explicit index, yet the sampling mechanism itself produces a kind of probabilistic indexing effect. From this viewpoint, one could develop index-based policies tailored to target detection or extend the BPS approach toward Bayesian optimization by incorporating prior distributions.

### 4.4. ε-Greedy (EG) Baseline (Action Selection with Controlled Exploration)

The greedy policy deterministically probes the maximum-belief cell and may therefore be sensitive to early belief peaks. To include a standard lightweight alternative that injects explicit exploration while preserving the belief-driven structure, we introduce an ε-greedy (EG) baseline. Let $b_{t}\in \Delta^{N-1}$ denote the belief vector at time t, and let the one-step predicted belief be $b_{t+1}^{\prime }=b_{t}T$, where T is the transition matrix. This prediction step is identical to those used in the greedy and BPS policies. We define the greedy action under the predicted belief as follows:

(13) $$s_{t+1}^{G}=\underset{i\in \{1,\ldots ,N\}}{argmax\;}b_{t+1}^{\prime }(i)$$

The ε-greedy action $s_{t+1}^{EG}$ is then defined by the following mixture rule:

(14) $$s_{t+1}^{EG}=\{\begin{array}{cc} s_{t+1}^{G} & \mathrm{with}\;\mathrm{probability}\;1-\varepsilon \\ \tilde{s}∼U(\{1,\ldots ,N\}) & \mathrm{with}\;\mathrm{probability}\;\varepsilon \end{array}$$

where U({1,…,N}) denotes the uniform distribution over the N grid cells and ε∈[0,1] controls the exploration rate. With probability ε, the action $s_{t+1}^{EG}$ is sampled uniformly from {1,…,N}. The EG baseline uses the same belief prediction and posterior update as the other belief-driven policies; the only difference lies in the action-selection rule above. Under the ideal sensor assumption used in the main experiments, a miss at the probed cell yields the same posterior-update (miss-update) rule as Equation (5); EG differs from greedy/BPS only in the action-selection rule in Equation (14).

## 5. Experimental Setup and Results

Section 5 summarizes the experimental protocol and the simulation-based evidence used to compare the proposed belief-driven heuristics. We first describe the simulation environment and default parameter settings (Section 5.1), then report quantitative performance comparisons across grid sizes (Section 5.2), and then analyze belief-state dynamics via heatmaps (Section 5.3). We further examine detection-time distributions and variability (Section 5.4) and finally provide a sensitivity study under a simple noisy-sensor model (Section 5.5).

### 5.1. Experimental Setup

We constructed a simulation environment to evaluate the performance of the target-detection algorithms. In the baseline setting, as defined earlier, the target performs a random walk on a one-dimensional grid of size N, and the sensor probes one slot at a time. In all experiments reported here, we deliberately restrict attention to the symmetric reflecting random walk of Section 3 in order to obtain a clean comparison of policies under a single, well-controlled motion model. In practice, one could instantiate T to encode biased motion, region-dependent speeds, or mode-switching behaviors (e.g., loitering versus rapid escape), and we expect the qualitative trade-offs between the greedy and belief-proportional sampling (probability-matching) policies to persist, although the absolute ETTD values would change. The main experimental parameters are summarized in Table 3; when necessary, some parameters were varied across experiments.

As shown above, we mainly used grid sizes N=10 and N=20 (with additional tests at N={5,10,15,20,25,30,35,40}). For each case, we ran four policies: the greedy policy, ε-greedy (EG), belief-proportional sampling (BPS), and a random baseline. We include ε-greedy as a lightweight exploration-controlled baseline that interpolates between purely greedy probing and uniform random probing without additional learning or model fitting. We designate the greedy policy as the primary competitive baseline, representing the standard myopic approach (a reasonable, simple alternative) for belief-based search. While simple deterministic patterns (e.g., systematic linear sweeps) exist, they are generally ill-suited for stochastic targets that can slip into previously scanned areas; thus, we focus on comparing belief-driven heuristics. The random policy (uniform slot selection) is included solely as a reference lower bound (chance-level performance) to contextualize the scale of ETTD, rather than as a competitive method. For every configuration, we performed 1000 independent simulations and measured average performance. Our primary metric was the ETTD, i.e., the expected number of steps required until detection. We also inspected how the belief distribution evolved over time and how each policy selected sensing locations. Unless otherwise stated, all experiments use the idealized binary detector with $P_{d}=1$ and $P_{fa}=0$; in Section 5.5, we additionally consider a noisy case with $P_{d}=0.8$ and $P_{fa}=0.02$ to examine the sensitivity of the policies to reduced detection probability.

**Figure 2:**
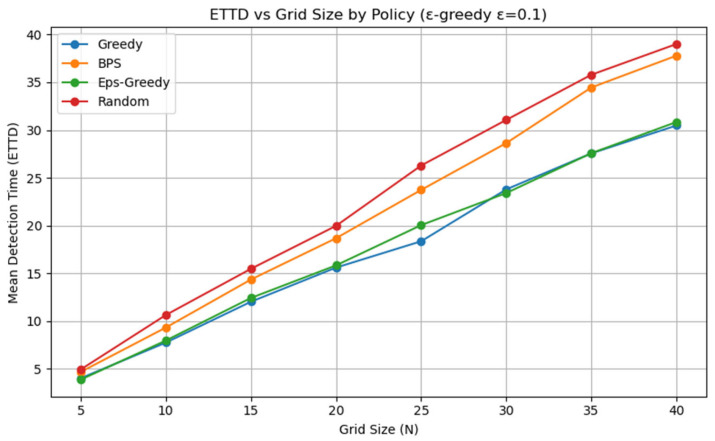
Average ETTD by policy versus grid size N under the ideal sensor model (Pd = 1, Pfa = 0), comparing greedy, BPS, ε-greedy (ε = 0.1), and random. Each point reports the mean over 1000 Monte Carlo episodes (seed = 42).

**Figure 3:**
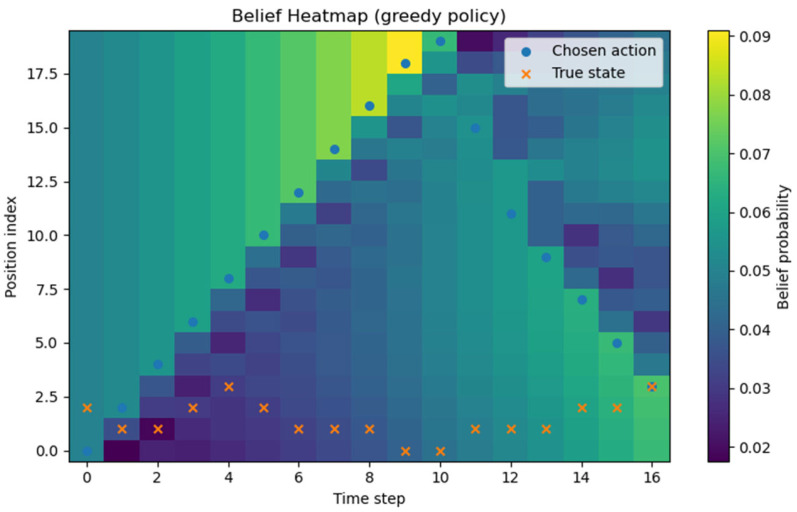
Greedy policy heatmap (belief and actions). Example episode at N=20. Color indicates belief probability $b_{t}(i)$ over grid position i(vertical axis) and time step t(horizontal axis, up to detection). Circle markers overlay the sensed slot s(t) chosen at each time step.

**Figure 4:**
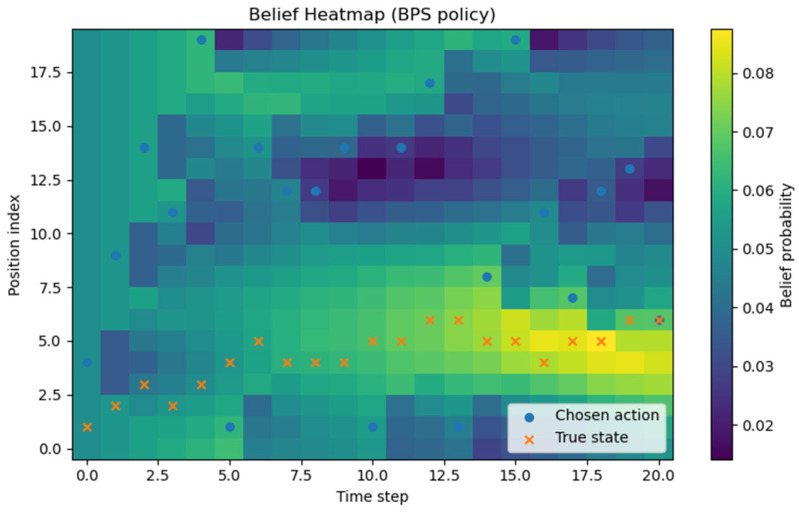
Belief-proportional sampling (BPS; probability-matching) policy heatmap (belief and actions). Example episode at N=20 visualized as $b_{t}(i)$ over position i and time t (up to detection). Circle markers indicate the sensing action s(t) selected at each time step.

**Figure 5:**
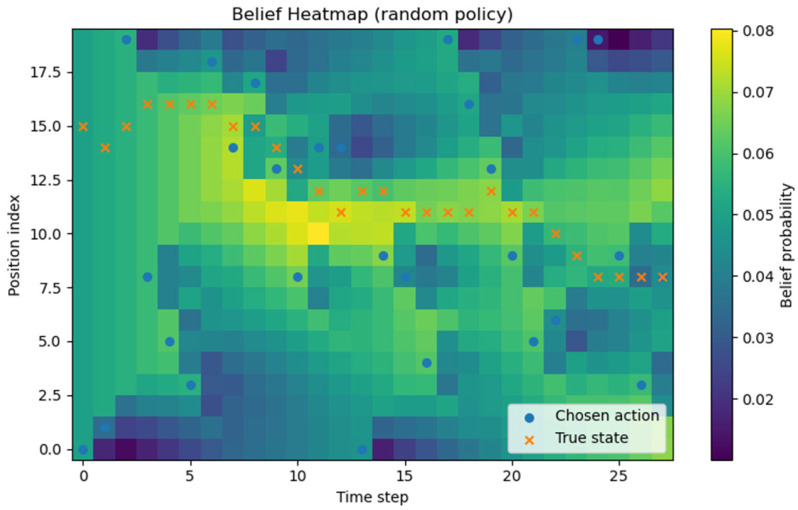
Random policy heatmap (belief and actions). Example episode at N=20 visualized as belief $b_{t}(i)$ over position i and time t(up to detection). Circle markers indicate the randomly selected sensing slot s(t) at each time step.

**Figure 6:**
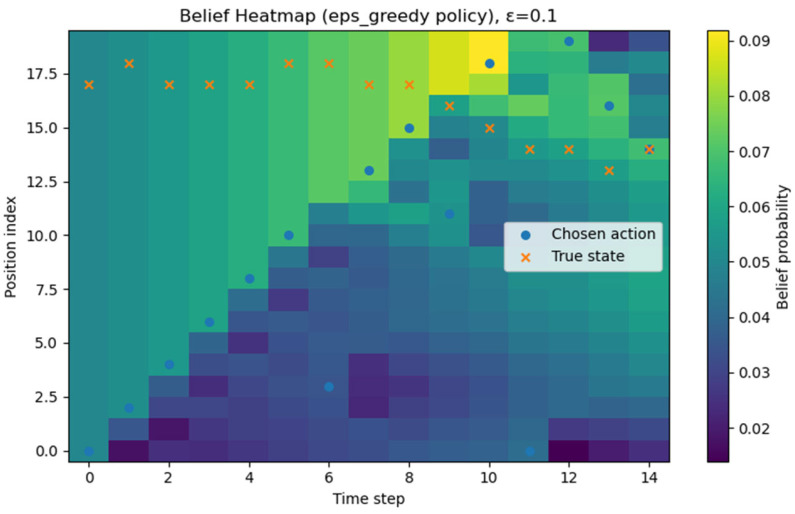
ε-greedy policy heatmap (belief and actions). Example episode at N=20 with ε=0.1. Color indicates the belief probability $b_{t}(i)$ over grid position i(vertical axis) and time step t (horizontal axis, up to detection). Circle markers overlay the sensed slot s(t) chosen at each time step. Cross markers indicate the true target state x(t) for reference.

**Figure 7:**
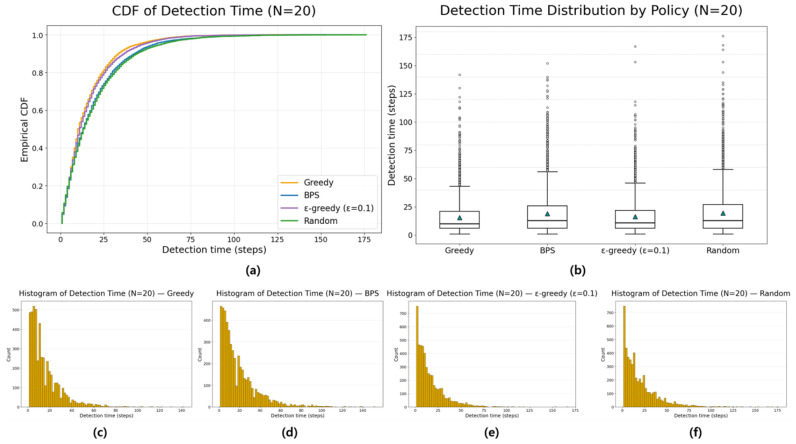
Detection-time distributions under four policies (N = 20). (**a**) Empirical cumulative distribution functions (CDFs) of detection time for greedy, belief-proportional sampling (BPS), ε-greedy (ε = 0.1), and random. (**b**) Boxplot comparison of detection-time distributions, where the center line denotes the median, the box spans the interquartile range (IQR), whiskers extend to 1.5 × IQR, circles indicate outliers, and the triangle marker denotes the sample mean. (**c**–**f**) Histograms of detection time for each policy. These plots visualize both central tendency and tail behavior, complementing the average ETTD comparisons reported in the main results.

**Figure 8:**
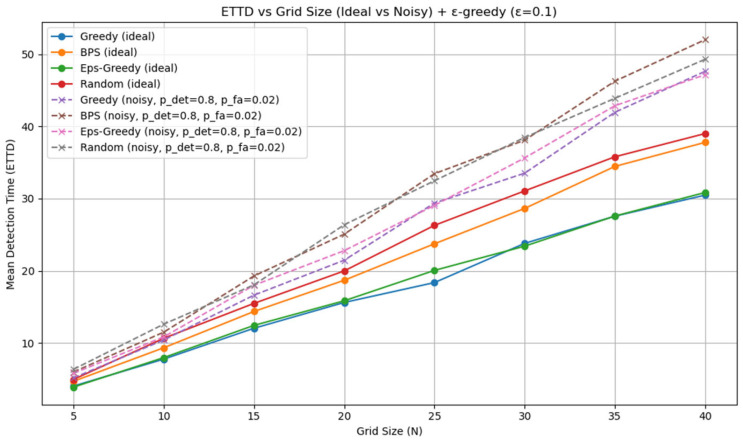
Mean termination time: under the ideal sensor it equals the time-to-true-detection, whereas under $\left(P_{d}=0.8,\;\;P_{fa}=0.02\right)$ it corresponds to time-to-first-alarm (true or false).

All experiments were implemented in Python (version 3.13.5), and each policy followed the definitions given earlier. Although stochasticity introduces run-to-run variance, averaging over about 1000 repetitions produced stable ETTD estimates, making the comparisons reliable. At this stage, however, the evaluation is purely simulation-based and does not include experiments on a real hardware platform. Consequently, the reported results should be interpreted as algorithmic performance under an idealized sensing model; assessing robustness to sensor noise, false alarms, and missed detections will require hardware-in-the-loop and field tests in future work. All figures and tables in this manuscript were generated by the authors from our original Monte Carlo simulations and analysis; no third-party figures, tables, or datasets are reproduced or adapted. All experiments and visualizations were generated by the authors from our own Monte Carlo simulations using Python (NumPy (version (2.1.3) for simulation and belief-state updates; Matplotlib (version 3.10.0) for plotting). For Figure 2, for each grid size N, we ran 1000 independent episodes and estimated ETTD as the sample mean of the resulting detection times; the plotted markers correspond to these discrete N settings, and the connecting lines are provided only as a visual guide. In Figure 3, Figure 4, Figure 5 and Figure 6, we visualize the belief evolution from a representative episode at N=20 by plotting the belief matrix $B\in R^{N\times T}$ with entries $B(i,t)=b_{t}(i)$, where the horizontal axis t denotes time steps within the episode (up to detection) and the vertical axis i denotes grid position. The overlaid circle markers indicate the sensing action s(t) selected at each time step. For Figure 7, we used the same Monte Carlo detection-time samples obtained at N=20 (1000 independent episodes per policy). The empirical CDF in Figure 7a was computed by sorting the detection times $\{T_{D}^{\left(m\right)}{\}}_{m=1}^{M}$ (with M=1000) and plotting ($t_{\left(k\right)},k/M$), where $t_{\left(k\right)}$ is the k-th order statistic. The box-and-whisker plot in Figure 7b was generated from the same samples using the standard quartile-based summary (median and interquartile range), with whiskers defined by the 1.5 × IQR rule and points beyond the whiskers shown as outliers. The histograms in Figure 7c–f were produced by binning the detection-time samples into integer-valued step counts and plotting the resulting frequencies (with identical binning logic across the four policies) to visualize the shape and tail behavior of the distributions. For Figure 8, we repeated the same Monte Carlo protocol as in Figure 2 under two sensing models: (i) the ideal sensor ($P_{d}=1$, $P_{fa}=0$) and (ii) a noisy sensor with missed detections and false alarms ($P_{d}=0.8$, $P_{fa}=0.02$). For each grid size N and each policy, we ran 1000 independent episodes and estimated the mean detection time (ETTD) as the sample mean of the recorded termination times. Solid (ideal) and dashed (noisy) curves connect these discrete sample means for visual guidance only. No third-party figures, tables, or datasets were reproduced or adapted. This study was conducted in a defense-related context, and the funding and security policy restrict the public release of source code and per-episode raw logs. To preserve reproducibility within these constraints, we provide an implementation-level specification of the environment dynamics, sensing model, belief update, and policy definitions (greedy, ε-greedy (EG; ε = 0.1), BPS, and uniform random), together with the complete Monte Carlo evaluation protocol. Moreover, all reported results were generated using a fixed pseudo-random seed of 42, enabling exact regeneration of the reported curves and summary statistics via independent clean-room reimplementation.

### 5.2. Performance Comparison

We first compared the average detection time (ETTD) of four policies, greedy, BPS, ε-greedy (ε = 0.1), and random, across grid sizes N ∈ {5, 10, 15, 20, 25, 30, 35, 40} under the ideal sensor assumption. Table 4 reports the mean ETTD (and standard deviation), and Figure 2 visualizes how ETTD scales with N. From the above results, the greedy policy achieved the lowest ETTD, indicating the highest efficiency. In the N=10 setting, it detected about 17% faster than BPS, and at N=20, it was again ahead by roughly 17%. Interestingly, at N=20, the average performance of BPS and the random policy appeared almost identical. This can be interpreted as a consequence of the target’s random-walk model used in our experiments: BPS exploration was sufficiently spread out on larger grids that its efficiency approached that of random probing. Compared with the greedy policy, BPS provides greater exploratory diversity, but this also incurs opportunity costs, so its overall average can fail to clearly outperform random. By contrast, the random policy finds the target with a fixed probability under any situation; however, lacking any advantageous strategy, it was consistently dominated by the greedy policy (e.g., by about 21% at N=20, based on Table 4).

Under the present random-walk target dynamics, EG closely tracks greedy in mean ETTD because the greedy action is selected with probability 1−ε at every step. Nevertheless, EG serves as a standard lightweight exploration-controlled baseline and can reduce sensitivity to early belief peaks in settings with higher model mismatch or partial observability. Figure 2 varies N over {5,10,15,20,25,30,35,40} and shows that the ETTD of all four policies grows roughly linearly with grid size. EG is nearly indistinguishable from greedy in this setting for ε=0.1, which is consistent with its mixture structure that selects the greedy action most of the time. For the greedy policy, doubling N nearly doubles the average detection time, indicating efficiency that scales proportionally with the search space even when the target moves. BPS and the random policy exhibit a similar upward trend, but with slightly steeper slopes than the greedy policy. This suggests that the relative advantage of the greedy policy increases as the space expands: the more candidate locations there are, the more effective it is to concentrate probes on the most promising slot. Each point in Figure 2 is the Monte Carlo estimate of $E[T_{D}]$ computed from 1000 independent episodes under the corresponding policy and grid size, and the plotted curves connect these discrete sample means to highlight overall scaling trends.

### 5.3. Analysis of Belief-State Dynamics

We visualized how the belief distribution and sensing actions evolve over time under each policy. Figure 3 illustrates, for one example scenario with N=20 using the greedy policy, a heatmap of the belief distribution and the chosen sensing locations over time. The horizontal axis denotes time, the vertical axis denotes grid position, and the color indicates the probability that the target is at that position. The blue circles s(t) mark the slot sensed by the agent at each time. Early on, the target’s location is unknown, and the belief is broadly distributed; as time progresses and observations accumulate, we see the belief mass concentrating toward specific directions.

Figure 3 visualizes, for each grid position, the belief probability (color intensity) together with the sensing slot chosen at each time (o). It clearly shows that the greedy policy consistently tracks the maximum of the predicted belief $b_{t+1}^{\prime }$. As time progresses, the high-intensity region of the heatmap (darker color) rapidly concentrates around a single location (a unique mode), and the o markers continuously follow this high-probability region. After a miss, the belief mass of the selected slot is removed and the remaining probabilities are renormalized (Bayesian update), causing a thin spread of belief to nearby slots; in the next prediction step, the probability flow of the transition matrix pulls the mass back to the core region, and this pattern repeats. As this process accumulates, just before detection, the belief becomes concentrated on essentially one slot, the selections also stabilize there, and the ETTD shortens rapidly. Consequently, the greedy policy exhibits clear advantages in terms of convergence speed, path consistency, and stability of variance.

In Figure 4, the characteristic randomized selection of belief-proportional sampling appears clearly. The o markers do not always stay on the single most likely (maximum) location; they sometimes move to relatively open surrounding areas. This is because the policy uses the belief distribution itself as the action probabilities and thus performs exploration. As a result, compared with the greedy policy, the heatmap shows more frequent cycles of partial spreading and re-concentration of belief, and it generally takes longer for the belief mass to collapse to a single peak. However, as observations accumulate over time, the influence of the transition dynamics becomes dominant, the high-probability region becomes distinctly shaped, and, just before detection, the pattern converges to a sharply peaked form similar to greedy. Overall, belief-proportional sampling tends to yield a larger average ETTD and variance than greedy, but its allowance for occasional suboptimal choices makes it more robust to nonstationarity and model mismatch.

In Figure 5, because the random policy’s actions are independent of the belief, the ○ markers are scattered with no relation to the high-intensity regions of the heatmap. Although the belief itself slowly concentrates into a particular area through Bayesian updating, the selections do not support that flow, so belief convergence is perceptibly slower, and more steps are required to achieve detection. Notably, even when the belief begins to concentrate, the policy continues to probe other locations at random, repeatedly undermining the efficiency of evidence accumulation. Consequently, among the four policies, the random policy exhibits the longest average expected time to detection and the largest variance, visually underscoring the need for belief-based policy design.

Figure 6 illustrates the time evolution of the predicted belief $b_{t+1}^{\prime }$ under the ε-greedy baseline. At each time step, the belief is propagated using the one-step Markov transition model and then used for action selection. The ε-greedy policy selects the maximum-belief cell with probability 1−ε, but with probability ε it samples a cell uniformly at random, producing occasional deviations of the chosen action (blue circles) from the locally dominant belief peak. The alignment (or mismatch) between the chosen action and the true target state (orange crosses) highlights how controlled exploration can mitigate over-commitment to early belief peaks, while still largely retaining the efficiency of greedy probing when ε is small.

### 5.4. Additional Analysis: Detection Success Probability and Variance

This section synthesizes the results, focusing on four aspects: the average detection time by policy, the temporal evolution of the belief state, and the distribution of detection times (variance and tail behavior). First, when we vary the grid size N and compare the mean ETTD of the four policies (greedy, BPS, EG, and random), the average detection time for all policies increases almost linearly with N. Among them, the greedy policy consistently achieves the lowest average detection time across the entire range, while BPS incurs a higher mean due to probabilistic exploration, and the random policy is uniformly the least efficient. This indicates that prioritizing the most promising slots—a strategy intrinsic to the greedy policy—is advantageous in expectation even when the target executes a random walk.

The visualizations of the belief state over time show that the greedy policy continually tracks the maximum of the predicted belief, causing the belief distribution to concentrate rapidly into a unimodal structure and the chosen slots to remain stably within the high-probability region. In contrast, BPS, which uses the belief as action probabilities, intermittently explores the surrounding region; as a result, cycles of spreading and re-concentration are observed more frequently, and the convergence of the belief is comparatively slower. Because the random policy acts independently of the belief, its selections are scattered across the heatmap, largely unrelated to the high-intensity region; consequently, belief convergence is the slowest, and more steps are required before detection. These qualitative patterns are consistent with the performance ranking observed in the average ETTD comparison.

Table 5 summarizes, for representative grid sizes N∈{10,20,30,40}, both detection-time performance and computational resource usage over 1000 Monte Carlo episodes per setting. The detection metric is the expected time to detection (ETTD), reported as mean ± standard deviation across episodes. “Time for 1000 eps (s)” is the measured wall-clock runtime required to complete the 1000-episode batch for each policy and N (measured on the same execution environment and with a fixed pseudo-random seed). “Total steps” denotes the sum of per-episode detection times, i.e., $\sum_{e=1}^{1000}T_{e}\approx 1000\times \mathrm{ETTD}\;\left(\mathrm{mean}\right)$, and therefore provides a direct link between the detection metric and the total amount of simulation work performed.

To quantify computational cost in a reproducible manner, we report an operation-count estimate for the dominant computation in our implementation: belief prediction via the matrix–vector multiplication b←bT. Under a dense transition-matrix implementation, this step requires approximately $N^{2}$ multiplications and N(N−1) additions per time step (≈$2N^{2}$ FLOPs). Accordingly, “Matvec FLOPs est.” is computed as follows:

(15) $$\mathrm{FLOPs}\approx 2N^{2}\times (\mathrm{Total}\;\mathrm{steps})$$

“ETTD × N” and “ETTD × $N^{2}$” are normalized cost proxies that reflect the expected total work under sparse/local (O(N)) and dense ($O(N^{2})$) belief-update implementations, respectively. These columns allow readers to compare policies in terms of both detection performance and computational burden and reproduce the cost calculations independently, even when source code and raw episode logs cannot be publicly released due to security restrictions.

Although all four policies share the same minimum detection time of one step, the mean and variance confirm the efficiency advantage of greedy (and closely, EG). Greedy concentrates effort on the maximum-belief cell, whereas EG follows greedy with probability 1−ε while injecting uniform exploration with probability ε. In contrast, BPS and random make stochastic selections that more frequently broaden the search path, increasing mean delay and tail risk.

Figure 7a reports the empirical CDF of detection time at N=20. The greedy policy curve is consistently left-shifted and rises earlier, indicating faster accumulation of detection probability over time, whereas BPS exhibits a slower rise and a heavier tail, and the random baseline is the slowest. Figure 7b complements this view by summarizing the distribution via a box plot: greedy achieves the smallest median and interquartile range, while BPS and random show broader dispersion and more frequent high-delay outliers, reflecting larger variance and tail risk. Finally, the histograms in Figure 7c–f provide policy-specific shape information; compared with greedy Figure 7c, BPS Figure 7d, EG Figure 7e, and random Figure 7f place more mass on longer detection times, visually confirming the delayed-case frequency that drives their higher mean/variance reported in Table 5.

In summary, under the present setting (random-walk target and unit per-step cost until detection), the greedy policy is the most efficient in minimizing the ETTD. The BPS policy does not match the greedy policy in average performance, yet its stochastic nature mitigates the risk of getting trapped in local belief peaks, a property that is theoretically advantageous under high uncertainty. The random policy serves as a baseline and trails the belief-based policies in both mean and variance. For practical deployment, the choice should balance efficiency (greedy) and robustness (BPS) according to the level of environmental uncertainty and the cost structure (e.g., penalties for repeated probing, heterogeneous sensing costs).

Finally, we briefly comment on computational cost and how it would scale in more complex environments. In the present 1D setting with grid sizes up to N=40, the dominant operations per time step are the belief prediction $b_{t+1}^{\prime }=b_{t}T$, and the subsequent selection/update of a single sensing slot, whose cost is bounded by $O(N^{2})$ for dense dynamics or O(N) for sparse, local motion models. Since the expected time to detection grows approximately linearly with N (Figure 2), the overall runtime of each episode also scales roughly linearly (sparse case) or quadratically (dense case) in the number of grid cells, which remains lightweight compared with exact POMDP solvers. In higher-dimensional workspaces, a 2D or 3D environment discretized into $N_{\mathrm{cells}}$ states would inherit the same structure: with local transition dynamics, the greedy and BPS policies still require only $O(N_{\mathrm{cells}})$ work per time step for belief propagation and action selection, whereas exact value iteration over the belief space would be computationally prohibitive. Thus, while a full feasibility study on large-scale 2D/3D maps is left for future work, the proposed heuristics are computationally well-suited as building blocks within larger search-and-tracking architectures. This mean–tail trade-off suggests different operating regimes for each policy. The greedy policy is well-suited when minimizing the ETTD is the primary objective and the belief/motion model is reasonably reliable, because it always prioritizes the highest-belief cell. In contrast, BPS can be preferable when robustness and risk control are important: by maintaining a nonzero probability of probing lower-belief cells, it mitigates over-commitment to early belief peaks and can reduce the likelihood of very long detection times. Practically, greedy is attractive for time-critical search with stable dynamics, whereas BPS can be preferable for persistent monitoring or settings with higher uncertainty/model mismatch, where avoiding rare but extreme delays is important.

### 5.5. Sensitivity to Sensor Noise with False Alarms

To further examine the robustness of the four policies under more realistic sensing conditions, we performed an additional experiment in which the binary detector was both noisy and prone to false alarms. In this setting, when the probed slot coincides with the true target position, a detection alarm is generated only with probability $P_{d}=0.8$; when the target is absent from the probed slot, a spurious alarm is generated with probability $P_{fa}=0.02$. As in the previous experiments, once an alarm is raised (whether true or false), the episode terminates and the corresponding detection time is recorded. For clarity, we refer to this termination metric as “time-to-alarm” in this subsection, since alarms may be spurious when $P_{fa}>0$. The target transition model and all remaining parameters follow Table 3. Figure 8 shows the mean detection time as a function of the grid size N for the ideal sensor (solid curves) and the noisy sensor with false alarms (dashed curves, $P_{d}=0.8$, $P_{fa}=0.02$). Introducing both missed detections and false alarms shifts the ETTD curves upward across all four policies, reflecting the additional uncertainty in the sensing process. However, the qualitative behavior of the policies is largely preserved. The greedy policy continues to yield the lowest mean detection time, while the random policy and BPS remain less efficient, with BPS suffering the most under this noise model due to its stronger exploratory behavior. The slopes of the noisy curves are similar to those of the ideal case, indicating that the increase in ETTD remains approximately linear in the grid size. Overall, these results suggest that the performance ranking of greedy, BPS, and random probing observed in the idealized setting is preserved even when moderate levels of both missed detections and false alarms are present.

## 6. Discussion

The ideal detector ($P_{d}=1,\;\;P_{fa}=0$) should be interpreted as an optimistic upper-bound baseline rather than a realistic sensing model. Under $P_{d}<1$, repeated misses slow evidence accumulation and typically increase ETTD, while $P_{fa}>0$ can distort termination statistics unless additional confirmation logic is introduced. The sensitivity study in Section 5.5 (Figure 8) is included to partially close this realism gap by quantifying how moderate missed detections and low-rate false alarms affect the performance trends. Before discussing the quantitative trade-offs between the two policies, it is important to clarify the scope of applicability. The simulation results in Section 5 provide direct evidence to answer the research questions posed in Section 1. For RQ1, the detection-time statistics and distributional plots show that the greedy policy consistently achieves a lower expected time to detection than belief-proportional sampling, while also revealing differences in variance and tail behavior. For RQ2, the empirical distributional comparisons highlight the exploitation–exploration trade-off: greedy concentrates sensing on the current belief peak, whereas BPS injects stochastic exploration that can improve robustness under uncertainty. For RQ3, the multi-N experiments quantify how the performance gaps scale with grid size, establishing a reusable quantitative baseline for future extensions to noisier sensors and higher-dimensional search. Our model deliberately omits many factors that are crucial in realistic defense, disaster-response, and surveillance-robotics deployments, such as 2D/3D geometry, occlusions, multi-sensor coordination, communication constraints, and hardware-level imperfections. The goal of this study is not to provide an end-to-end field-ready system, but rather to analyze, in isolation, a canonical “linear search under limited sensing resources” motif that can appear as one component within larger planning architectures. Accordingly, the results should be interpreted as providing conceptual insight and quantitative baselines for such subproblems, while bridging the gap to full-scale applications will require richer motion models, sensor noise, and hardware-in-the-loop validation in future work. For instance, the current equal-probability random-walk assumption does not capture many realistic behaviors such as drift along road networks, environment-dependent stopping patterns, or adversarial avoidance of sensed regions; extending the proposed policies to such structured motion models is a key step toward operational deployment. In particular, a more realistic validation pipeline would couple the proposed policies with physical sensors (e.g., radar, lidar, or vision-based detectors) and systematically inject measurement noise, false positives, and missed detections in order to quantify how the belief-update mechanism and ETTD degrade under non-ideal conditions. Moreover, the present simulations employ an idealized binary detection/miss model (Table 3) without explicit detection and false-alarm parameters; extending the framework to imperfect sensors with specified Pd and Pfa values is an important direction for making the results more representative of real-world sensing systems.

Synthesizing the experimental results, the proposed greedy policy achieved the lowest ETTD and thus favored faster detection, which aligns with intuition from classical search theory. However, the greedy policy risks repeatedly probing an incorrect slot when the belief estimate is wrong, and its performance can degrade sharply if the target’s motion model changes or the environment is noisy. By contrast, the BPS policy explores diverse slots in the early stages, offering the possibility of recovery from misspecified beliefs and exhibiting greater robustness to environmental variability. While our current linear-grid experiments focused on varying grid sizes and sensor noise, the literature suggests that such probabilistic exploration can offer the possibility of recovery from misspecified beliefs [27]. In field settings where the target’s motion characteristics are unknown or highly variable, the probabilistic nature of BPS can be advantageous.

To understand the trade-off between the two proposed policies (greedy and BPS) more structurally from the standpoint of restless MABs, note that the greedy policy reduces the search space by always selecting the slot with the highest belief, whereas BPS balances exploration and exploitation in a probabilistic manner [28]. Although the Whittle-index policy [24] can be near-optimal in theory, its computational complexity makes real-time, online deployment difficult. In addition, Tong et al. [29] proposed a federated-learning-based distributed RMAB framework to address multi-agent cooperation.

Future work could leverage such learning-based approaches to approximate the Whittle index or to extend the problem to more complex settings, such as two-dimensional grids and multiple targets. In particular, with multiple targets, the problem can be generalized to a multi-bandit formulation by maintaining independent beliefs per target, and, following cooperative UAV search studies [10], information sharing among multiple agents could further improve search efficiency. Beyond one-dimensional environments, generalizing to two-dimensional grids or real terrain would be valuable for practical applicability. In multi-dimensional grids, a naive extension of the greedy policy would still perform an O(N) argmax over all cells, but the curse of dimensionality manifests through increasingly fragmented belief landscapes and a proliferation of local maxima. As a result, a direct greedy rule may prematurely focus on a single mode of the belief map and neglect competing hypotheses unless it is combined with spatial hierarchy (e.g., sector-based or scanline-based decomposition), receding-horizon planning, or stochastic exploration components. A promising direction is to treat the one-dimensional policies studied in this paper as primitives that operate along individual tracks or sectors within a larger 2D/3D workspace, while higher-level planners coordinate between tracks to manage both computational load and the risk of local optima. In particular, a natural next step is to instantiate these policies on discretized 2D and 3D grids and quantitatively test whether the relative efficiency and robustness trends observed in our one-dimensional setting persist under more realistic geometric layouts, sensing footprints, and operational constraints.

Overall, Section 5 and Section 6 establish consistent empirical evidence and an interpretable mechanism-level explanation for the observed trade-offs between exploitation-driven and exploration-driven sensing. In particular, the results clarify when a belief-argmax rule is advantageous for minimizing expected time to detection and when stochastic belief-proportional sampling can offer robustness against misspecification and uncertainty. These findings, together with the stated modeling limitations, position the present study as a quantitative baseline rather than a field-ready solution. We therefore conclude by consolidating the answers to the research questions, the added value of the benchmark, and the practical challenges that arise when extending the framework to noisier sensors and higher-dimensional deployments.

## 7. Conclusions

This study proposed and evaluated reinforcement-learning-inspired, bandit-style heuristic sensing policies for moving target detection in a partially observable one-dimensional linear-grid environment with binary observations. We focused on training-free decision rules defined directly on the belief state: a greedy belief-argmax heuristic (RMAB-inspired; not a Whittle-index policy) and a belief-proportional sampling (probability-matching) policy, with an ε-greedy baseline (ε = 0.1) and a uniform random policy for reference. Our simulation results demonstrate that the greedy policy consistently achieves a lower mean ETTD across various grid sizes compared to the baselines. The belief heatmaps offer a mechanistic interpretation of policy behavior: greedy sensing concentrates measurements near the belief peak, whereas BPS injects stochastic exploration that can reduce persistent fixation on incorrect hypotheses under uncertainty. Finally, multi-N comparisons quantify how performance gaps scale with problem size, establishing a reusable quantitative baseline for subsequent studies. Overall, the added value of this paper is an analytically transparent and computationally lightweight benchmark that can be executed online without a training phase. Practical implementation challenges arise when moving beyond the idealized setting, including sensitivity to transition-model mismatch, increased computational burden in higher-dimensional deployments, and degradation under missed detections and false alarms. Future work will extend the framework to multiple targets and higher-dimensional grids, incorporate richer sensor/target models, and evaluate learning-based approaches that learn compact belief representations to test whether the observed trends persist in noisier deployments.

## Figures and Tables

**Table 1:** Summary of representative related works and the gap addressed by this paper.

Category/Representative Works	Advantages	Limitations/Gap (w.r.t. This Study)
Classical search/planning for static or Markovian targets (e.g., [1,7])	Clear principles for efficient sequential search; often analytically grounded	Often assumes known distributions/plans; limited focus on online belief-driven sensing with moving targets in POMDP form
Dynamic hypothesis testing/resource allocation (e.g., [2])	Connects search with decision-theoretic testing; principled stopping/selection logic	Typically not framed as a lightweight benchmark focused on ETTD under a simple reflecting random-walk model
Robotic pursuit/search with known maps (e.g., [9])	Considers realistic constraints and multi-robot coordination	Planning is NP-hard; solutions are often approximation-heavy and not designed as a minimal analytic baseline
Cooperative Bayesian UAV search/tracking (e.g., [10,11])	Real-time Bayesian belief updates for multi-agent settings	Emphasis on system-level deployment; less emphasis on isolating policy trade-offs in a minimal 1D benchmark
Point-based POMDP solvers (e.g., [19,20,21,22,23])	Systematic approximations to POMDP optimal policies	Still computationally heavy; less “closed-form/training-free/purely online” as a baseline
Deep RL for POMDP-style search (e.g., [3,12,13])	Handles complex 2D/3D and multi-agent settings; strong empirical performance	Requires training, tuning, and large compute; reduced interpretability; difficult to use as a transparent baseline
Bandit/restless multi-armed bandit (RMAB)/Thompson sampling theory (e.g., [24,25])	Provides exploration–exploitation framework; interpretable decision rules	Direct application to belief-based moving-target detection is often nontrivial; motivates heuristic, lightweight policies
This paper	Training-free, purely online heuristics; explicit belief-MDP formulation; quantitative ETTD comparison across grid sizes	Stylized 1D benchmark (intended as a baseline for future 2D/3D extensions)

**Table 2:** One-step transition probabilities of the target.

Current Position i	Distribution of Next Position $P\left(X_{t+1}=j|X_{t}=i\right)$	
2≤i≤N−1 (interior)	$P\left(X_{t+1}=i-1\right)=P\left(X_{t+1}=i\right)=P\left(X_{t+1}=i+1\right)=\frac{1}{3}$	(1)
i=1 (left boundary)	$P\left(X_{t+1}=1\right)=\frac{2}{3},\;P\left(X_{t+1}=2\right)=\frac{1}{3}$	(2)
i=N (right boundary)	$P\left(X_{t+1}=N\right)=\frac{2}{3},\;P\left(X_{t+1}=N-1\right)=\frac{1}{3}$	(3)

**Table 3:** Simulation parameters.

Parameter	Value (Default)	Description
Grid size N	5~40	Number of slots N
Initial target distribution	Uniform	$\mathrm{Prior}\;\mathrm{belief}\;b_{0}$
Target motion model	Transition probabilities in Table 2	Random walk with boundary behavior (stay/left/right)
Sensor/observation model	Idealized binary detector (Pd = 1, Pfa = 0)	At the chosen slot, observe presence (o = 1) or absence (o = 0); see Section 3 for discussion
Policies (algorithms)	Greedy (belief-argmax), BPS (belief-proportional sampling), ε-greedy (ε = 0.1), Random (uniform slot selection)	Baselines: RMAB-inspired heuristic greedy; Belief-proportional sampling; ε-greedy (ε = 0.1); uniform random
Number of episodes	1000 per setting	Independent simulation runs per scenario
Stopping condition	Stop upon detection; otherwise, force stop at 1000 steps	Episode termination rule
Random seed	42	Fixed to 42 for all runs (Monte Carlo + figures).
Exploration rate ε (EG only)	0.1	Probability of uniform exploration in ε-greedy action selection

**Table 4:** Average detection time (ETTD) by policy.

Grid Size N	RMAB-Inspired Heuristic (Greedy)	Belief-Proportional Sampling (BPS; Probability-Matching)	ε-Greedy Policy	Random Policy
10	~7.7 steps	~9.3 steps	~7.9 steps	~10.6 steps
20	~15.6 steps	~18.6 steps	~15.8 steps	~19.9 steps
30	~23.7 steps	~28.6 steps	~23.4 steps	~31.0 steps
40	~30.4 steps	~37.7 steps	~30.8 steps	~39.0 steps

**Table 5:** Summary of detection-time statistics and computational time/cost (1000 episodes per setting).

Policy (N)	ETTD (Mean ± std)	Time for 1000 Eps (s)	Total Steps	Matvec FLOPs est.	ETTD × N	ETTD × N^2^
Greedy (10)	7.750 ± 7.198	0.078	7750	1,550,000	77.50	775.0
BPS (10)	9.324 ± 8.569	1.146	9324	1,864,800	93.24	932.4
EG (10)	7.962 ± 6.834	0.082	7962	1,592,400	79.62	796.2
Random (10)	10.637 ± 9.923	0.087	10,637	2,127,400	106.37	1063.7
Greedy (20)	15.603 ± 14.207	0.158	15,603	12,482,400	312.06	6241.2
BPS (20)	18.678 ± 17.619	0.293	18,678	14,942,400	373.56	7471.2
EG (20)	15.836 ± 15.857	0.167	15,836	12,668,800	316.72	6334.4
Random (20)	19.974 ± 19.531	0.161	19,974	15,979,200	399.48	7989.6
Greedy (30)	23.794 ± 22.876	0.243	23,794	42,829,200	713.82	21,414.6
BPS (30)	28.639 ± 27.796	0.463	28,639	51,550,200	859.17	25,775.1
EG (30)	23.410 ± 22.420	0.251	23,410	42,138,000	702.30	21,069.0
Random (30)	31.066 ± 31.157	0.252	31,066	55,918,800	931.98	27,959.4
Greedy (40)	30.484 ± 28.563	0.334	30,484	97,548,800	1219.36	48,774.4
BPS (40)	37.793 ± 34.202	0.600	37,793	120,937,600	1511.72	60,468.8
EG (40)	30.847 ± 28.217	0.322	30,847	98,710,400	1233.88	49,355.2
Random (40)	39.013 ± 38.739	0.317	39,013	124,841,600	1560.52	62,420.8

## Data Availability

Due to funding and security restrictions associated with defense-related research, the source code and per-episode raw simulation logs cannot be publicly released. To support reproducibility, the manuscript provides an implementation-level specification of the environment, sensing model, belief update, and policies, along with the complete Monte Carlo evaluation protocol. All experiments were executed with a fixed pseudo-random seed (42) to facilitate exact replication via independent clean-room implementation.

## Abbreviations

**Abbreviation**	**Full Term**	**Brief Note**
ETTD	expected time to detection	Primary metric
POMDP	partially observable Markov decision process	Belief-MDP formulation
MDP	Markov decision process	Reference framework
MAB	multi-armed bandit	Bandit baseline concept
RMAB	restless multi-armed bandit	Conceptual reference
BPS	belief-proportional sampling (probability matching)	Belief-proportional sampling policy
RL	reinforcement learning	Used in “RL-inspired” context
PMF	probability mass function	Discrete distribution over slots
PDF	probability density function	Continuous-variable distribution
UAV	unmanned aerial vehicle	Example application platform
Pd	probability of detection	Sensor parameter
Pfa	probability of false alarm	Sensor parameter
CDF	cumulative distribution function	Detection-time distribution plot
DRQN	deep recurrent Q-network	Related work (recurrent deep RL)
A3C-LSTM	asynchronous advantage actor–critic with long short-term memory	Related work (deep RL)
PPO	proximal policy optimization	Related work (deep RL)
SARSOP	Successive Approximations of the Reachable Space under Optimal Policies	Point-based POMDP planner
EG	epsilon-greedy (baseline)	Exploration–exploitation mixture baseline (greedy with prob. 1−ε, uniform exploration with prob. ε)
